# Impact of social activity restriction and routine patient screening as a preventive measurement for tertiary referral hospital staff in a country with high COVID-19 incidence

**DOI:** 10.1016/j.ijregi.2021.11.007

**Published:** 2021-11-26

**Authors:** Riyadi Adrizain, Siti Jubaedah, Eva Nursanty Fitriany, Rudi Wicaksana, Yovita Hartantri, Delita Prihatini, Dewi Kartika Turbawati, Basti Andriyoko, Ahmad Ramdan, Iwan Abdul Rachman, Melati Sudiro, Lina Lasminingrum

**Affiliations:** 1 Infection and Control Committee Dr. Hasan Sadikin General Hospital, Bandung West Java, Indonesia; 2 Department of Child Health, Department of Pediatrics, Faculty of Medicine Universitas Padjadjaran/Dr. Hasan Sadikin General Hospital, Bandung, Indonesia; 3 Emerging and Re-emerging Infectious diseases Team Hasan Sadikin General Hospital/Department of Internal Medicine, Faculty of Medicine Universitas Padjadjaran, Bandung West Java, Indonesia; 4 Department of Clinical Pathology, Faculty of Medicine Universitas Padjadjaran, Bandung West Java, Indonesia; 5 Directorate of Human Resource Education and Research Hasan Sadikin General Hospital Bandung, West Java, Indonesia; 6 Directorate of Medical and Nursing Services Hasan Sadikin General Hospital/Department of Otorhinolaryngology-Head and Neck, Bandung West Java, Indonesia

**Keywords:** COVID-19, Delta variant, hospital staff, patient caregiver, routine screening, social activity restriction

## Abstract

**Background:**

Measuring COVID-19 incidence among hospital staff and the influencing factors and preventative measures affecting outcomes is important given their high risk of exposure and potential impacts on health service provision.

**Method:**

Study participants included all hospital staff with COVID-19 confirmed by real-time reverse transcription-polymerase chain reaction (RT-PCR) from March 2020 to July 2021. Data were collected on age, gender, occupation, working area, symptoms and vaccination status. We also collected data on pediatric oncology patients and their caregivers to review the hospital screening policy.

**Results:**

Approximately 59% of positive cases among hospital staff occurred in the green zone; 75% were fully vaccinated. Whole-genome sequencing indicated that staff infections in June 2021 were Delta variant. A decrease in cases coincided with government implementation of social activity restriction. When RT-PCR was performed in suspected cases, 3 of 36 pediatric oncology patients and 10 staff tested positive. After routine screening, 8 of 121 patients, 3 patient caregivers, and 5 staff tested positive, all were asymptomatic, and all were infected in the community

**Conclusions:**

Routine testing for staff, patients and caregivers, vaccination booster programs, continuing education of health care workers, and government policy, such as social activity restriction, are needed to protect frontline workers.

## Highlights

• Health care workers are a high risk population for COVID-19 reinfection
• Vaccination breakthrough cases found after the mass vaccination program
• Social activity restriction is effective in lowering COVID-19 case numbers
• Routine screening for all patients recommended for a safe working environment

## Introduction

COVID-19 is an infection caused by SARS-CoV-2, with high transmission. The virus has spread so rapidly that, by 31 July 2021, almost 200 million people had been infected, and more than 4.2 million had died within 1.5 years (Abohamr et al., 2020). With 3 409 658 cases and 94 119 deaths as of 31 July 2021, Indonesia ranks as the fifth country in the world for total COVID-19 infections and twelfth for total deaths (Alexandar et al., 2021, Andriani, 2020, Borchering et al., 2021). Among those infected, worldwide 11%–29% cases were health care workers (HCW) (Bracis et al., 2021, Bradshaw et al., 2021, Burrer et al., 2020). HCWs have a 7-fold increased risk of contracting COVID-19 compared with other professions (Chung et al., 2021). People who work in hospitals have a higher exposure with patients and the general public (EMG - Transmission Group 2021), including being exposed to many patients with unknown COVID-19 status (Gitiyarko, 2021, Iavicoli et al., 2021). COVID-19 being asymptomatic in some people puts HCWs at higher risk (Bradshaw et al., 2021).

Indonesia has the highest COVID-19 positivity rate worldwide, with 1 out of 2 people testing positive (Jagadeesh Kumar et al., 2021). Studies in other countries have shown that social activity restriction (SAR) through lockdown is effective in lowering the number of daily COVID-19 cases (Jarvis et al., 2021, Karlsson and Fraenkel, 2020, Kementerian Kesehatan Republik Indonesia 2020, Kementerian Kesehatan Republik Indonesia 2021). Some public services, including hospitals, have used preventive measurements such as work-from-home or work-from-office policies and routine screening for their employees. Routine screening for all patients admitted to hospital to make the working environment safe for HCWs is still contentious, with cost-effectiveness being a key concern. However, the safety of HCWs is the main priority to keep the health system working well.

There are limited published studies on COVID-19 prevention measures focusing on social activity restriction and routine testing for in-patients and how these could affect the safety of hospital staff, especially in Indonesia, a country with one of the highest incidences of COVID-19 cases in the world. The objective of our study is to describe the characteristics of COVID-19 among hospital staff, factors that might influence the infection course and potential prevention measures.

## Methods

### Setting

We conducted a descriptive cross-sectional study in Hasan Sadikin General Hospital (HSGH), Bandung, from March 2020 to July 2021. HSGH is a tertiary referral center for COVID-19 in Indonesia with 944 beds and approximately 3000 staff, including physicians, nurses, midwives, pharmacists, radiographers, analysts and other operational staff. HSGH applied policies to prevent COVID-19 infection among staff, including work-from-home and work-from-office policies, supply of personal protective equipment, dividing the working area into red, yellow, and green zones, limiting the mobility of staff to go out of town or take some days off during holidays, and routine COVID-19 screening.

The first case of COVID-19 was reported in Indonesia in March 2020, and case numbers kept rising (Peraturan Presiden Republik Indonesia 2018). Numerous policies were implemented to control the pandemic. Unlike countries that applied lockdowns, Indonesia chose large-scale social restriction, or *Pembatasan Sosial Berskala Besar* (PSBB) in Bahasa, which restricted activities that might cause crowds and increase the risk of COVID-19 infection across school, workplace, physical worship and social-cultural activities. Other public policies were announced following PSBB, including Transitional PSBB from 12 to 25 October 2020, Public Activity Restriction (PAR), (or *Pemberlakuan Pembatasan Kegiatan Masyarakat* (PPKM) in Bahasa) from 11 January to 8 February 2021, Micro PAR from 9 February to 20 July 2021, and Emergency PAR from 3 to 25 July 2021 (Sinuhaji, 2021). The policies applied in Indonesia had different terminologies but were all applied in the same manner. Lockdown is a term used to describe the closure of an area so that no one can enter or leave to contain the infection and prevent transmission from one area to another. Social restriction is a term used in Indonesia to describe a restriction in public activity with the potential for higher COVID-19 infection risk (Sinuhaji, 2021).

We also compare the number of staff infected with COVID-19 in HSGH with national data during the same period. In addition, we included data from pediatric oncology patients and their caregivers who underwent real-time reverse-transcription polymerase chain reaction (RT-PCR) testing when COVID-19 was indicated and then when routinely screened, from 1 July 2020 to 8 January 2021.

### Participants

All HSGH staff with COVID-19 infection confirmed by RT-PCR with a cycle threshold of 11−40 were included in the study. Those who had symptoms of COVID-19 who were positive by antigen test but not from RT-PCR were excluded. All pediatric oncology patients admitted to HSGH and their caregivers who had RT-PCR tests were also included as study participants.

### Data collection and analysis

Data were collected on age, gender, occupation, working area, symptoms and vaccination status. We followed up on patient condition until they were discharged or died, whichever happened first. Patients were considered discharged when they had completed 14 days of self-quarantine with 3 days of no symptoms or 2 negative RT-PCR results (Kementerian Kesehatan Republik Indonesia 2021). For comparison, national data on COVID-19 infections were obtained from https://covid19.go.id/.

We use the disease severity classification published by the World Health Organization. Those with COVID-19 symptoms without evidence of viral pneumonia or hypoxia were considered mild cases. Those who had cough, dyspnea or fast breathing but no sign of severe pneumonia and saturated oxygen (SpO_2_) >90% were considered moderate cases. Those who had clinical signs of severe pneumonia with respiratory rate of >30 breaths per minute or signs of respiratory distress or reduced SpO_2_ <90% in room air were considered severe cases. Those who had signs of acute respiratory distress syndrome or sepsis, or septic shock, were considered to have critical disease. (Liu et al., 2021)

HSGH staff work in different areas divided into 3 zones according to COVID-19 transmission risk: the red zone is high risk places (emergency ward, COVID-19 ward, infection ward), yellow is medium risk (radiology unit, laboratory unit, pharmacy) and green is low risk (office, non-COVID-19 ward).

The COVID-19 vaccine program in Indonesia commenced in January 2021 using 2 doses of Synovac vaccine 14 days apart, with HCWs prioritized. Several vaccines were available during the study, including Synovac, Pfizer and Astra Zeneca; however, the government's free vaccination program only administered Synovac to the population. To 31 July 2021, 47 226 514 people had received the first dose, and 20 534 823 had received both doses. Our study categorized participants into unvaccinated, partially vaccinated and fully vaccinated groups. The unvaccinated group were those who had not been vaccinated, the partially vaccinated group were those who had received only the first dose, and the fully vaccinated group were those who had received both doses and at least 2 weeks had passed since the last dose (Mousten-Helms et al., 2021). The number of staff infected was counted weekly and monthly and compared with the national case report for the same week and month with a 1:1000 scale to compare the curve.

The HSGH clinical pathology laboratory carried out whole-genome sequencing among HCWs infected in June 2021 to identify the COVID-19 variant, particularly because of emerging new variants reported nationwide since May 2021. We also assessed those who were reinfected during the study period. Data were analyzed descriptively using Microsoft Excel.

We also collected pediatric oncology patients admitted to HSGH between 1 July 2020 and 8 January 2021 as representative data for patient screening policies because pediatric patients need a caregiver when admitted to the hospital. The data consists of the number of patients and their COVID-19 status from RT-PCR testing. We differentiated the data between the period at the beginning of the pandemic when RT-PCR was performed when COVID-19 was indicated and later, from October 2020, when RT-PCR was used in routine screening of all pediatric oncology patients and their caregivers admitted to HSGH.

The study has been approved by the ethics committee of HSGH, and parental permission was obtained for pediatric patients.

## Results

### Weekly cases

There were 1641 confirmed cases of COVID-19 in HSGH staff from March 2020 to July 2021. Characteristics of HCWs infected are listed in Table 1. Most of the staff infected are women (53%), aged 31−40 years (48%). Nurses are the profession with the highest number of COVID-19 cases. Disease severity ranged from mild to critical. Those with severe and critical disease were admitted to several hospitals within Bandung. Mild disease accounted for the highest number of cases (1622 cases, 99%), and 1635 (99.6%) cases were discharged. The majority of those infected were fully vaccinated (75%).

**Table 1:** Characteristic of Covid-19 among Staff in Hasan Sadikin General Hospital

	Vaccinated		Unvaccinated		Total	
	n=1233	75%	408	25%	1641	100%
Sex						
Male	611	50%	162	40%	773	47%
Female	622	50%	146	36%	868	53%
Age						
21-30	244	20%	100	25%	344	21%
31-40	622	50%	169	41%	791	48%
41-50	253	21%	92	23%	345	21%
>50	114	9%	47	12%	161	10%
Occupation						
Analyst	3	1%	6	1%	9	1%
Midwives	28	2%	2	1%	30	2%
Doctors	332	27%	163	40%	495	30%
Nurses	369	30%	139	34%	508	31%
Pharmacists	61	5%	15	4%	76	5%
Physiotherapist	9	1%	1	1%	10	1%
Nutritionist	26	2%	15	4%	41	2%
Non-medic	378	31%	65	16%	443	27%
Working area						
Green zone	757	61%	210	51%	967	59%
Yellow zone	365	30%	163	40%	528	32%
Red zone	111	9%	35	9%	146	9%
Disease severity						
Mild disease	1214	97%	390	95%	1622	95%
Moderate disease	14	2%	12	3%	26	3%
Severe disease	5	1%	3	1%	8	1%
Critical disease	0	0%	3	1%	3	1%
Management						
Self-quarantine	1180	96%	387	95%	1567	4%
Hospital Admission	53	4%	21	5%	74	96%
Outcome						
Discarded	1229	99%	406	99%	1635	99%
Dead	4	1%	2	1%	6	1%

**Figure 1:**
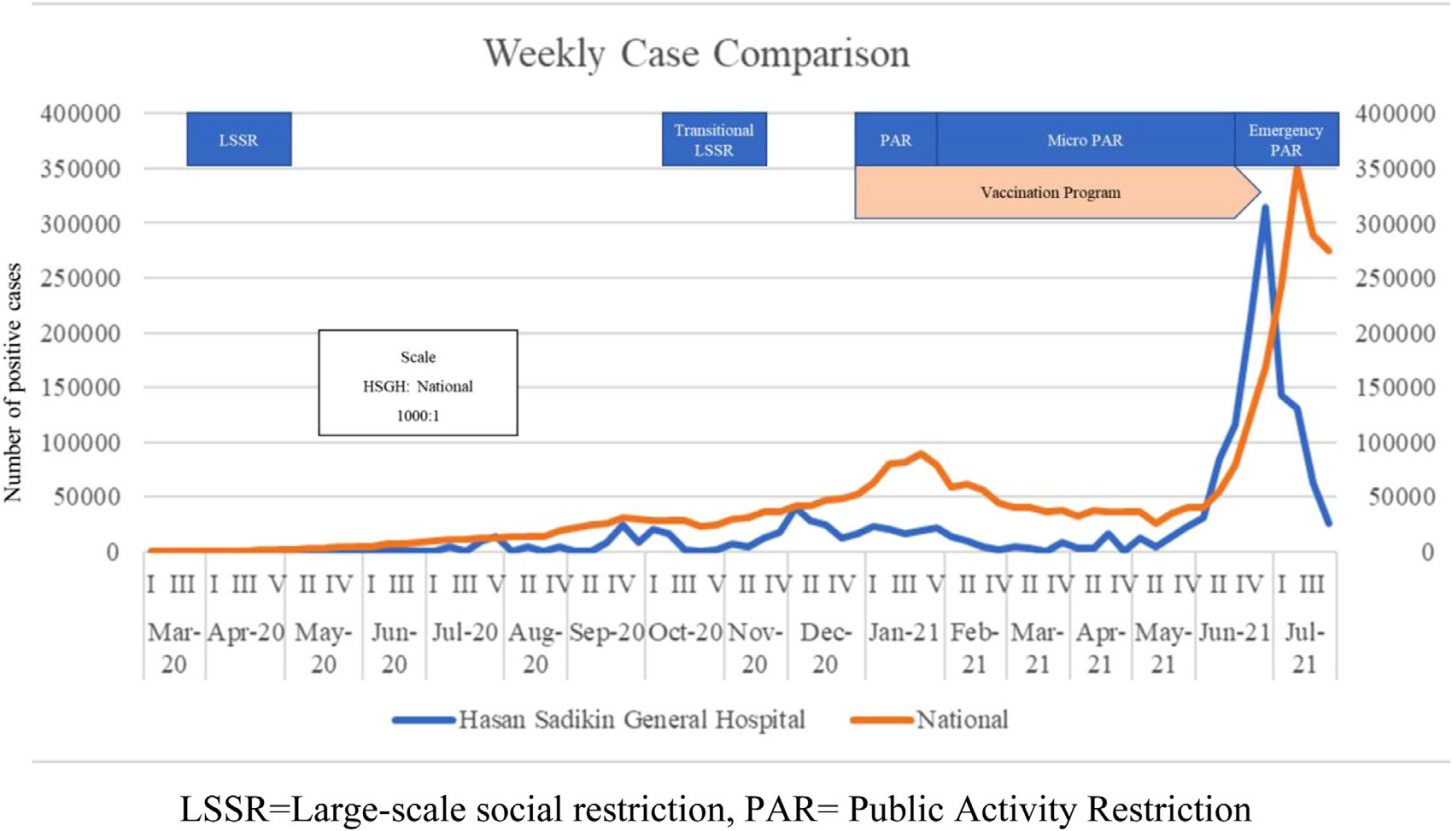
Weekly Case Comparison

A weekly case comparison of HSGH and national data is shown in Figure 1. In general, the curve is not parallel, with fluctuations in the HSGH curve and several peaks compared with national data, which showed a steady increase and only 2 peaks. The monthly COVID-19 positivity rate at HSGH was 12% in January 2021, and then down to 5% in February, before slowly increasing, 7% in March, 8% in April, 14% in May, and then surging to 53% in June before decreasing to 37% in July 2021. Whole-genome sequencing analysis in June 2021, during the second peak of incidence in Indonesia, revealed that all 19 infected HSGH staff had the Delta variant.

Of 1641 staff infected from March 2020 to July 2021, 28 (1.7%) staff had reinfection. In these cases, doctors and nurses were prevalent, with 46% and 43% of cases, respectively. Most were women (65%), aged 31−40 (60%); all but 1 had mild disease (96%); 38% worked in the yellow zone and 43% in the red zone; 50% were fully vaccinated and 50% unvaccinated (Table 2).

**Table 2:** Covid-19 Reinfection among Hospital Staff

	Vaccinated		Unvaccinated		Total	
	n=14	50%	n=14	50%	28	100%
Sex						
Male	7	50%	3	21%	10	36%
Female	7	50%	11	79%	18	64%
Age						
21-30	4	29%	2	14%	6	21%
31-40	6	43%	11	79%	17	61%
41-50	4	29%	1	7%	5	18%
Occupation						
Analyst	0	0%	0	0%	0	0%
Midwives	0	0%	0	0%	0	0%
Doctors	6	43%	7	50%	13	46%
Nurses	6	43%	6	43%	12	43%
Pharmacists	0	0%	0	0%	0	0%
Physiotherapist	1	7%	0	0%	1	4%
Nutritionist	0	0%	0	0%	0	0%
Non-medic	1	7%	1	7%	2	7%
Working area						
Green zone	8	57%	7	50%	15	54%
Yellow zone	6	43%	6	43%	12	43%
Red zone	0	0%	1	7%	1	3%
Disease severity						
Mild disease	14	100%	13	93%	27	96%
Moderate disease	0	0%	0	0%	0	0%
Severe disease	0	0%	1	7%	1	4%
Critical disease	0	0%	0	0%	0	0%
Management						
Self-quarantine	13	93%	13	93%	26	93%
Hospital Admission	1	7%	1	7%	2	7%
Outcome						
Discarded	14	100%	14	100%	28	100%

### Effect of social activity restriction and vaccination program

The different SAR policies in Indonesia led to varied results in lowering COVID-19 case numbers. Figure 1 shows a plateau during the first months of COVID-19 when PSBB and Transitional PSBB were applied. There was a rising case pattern at the end of 2020 and the beginning of 2021 when policies were relaxed. Along with the introduction of PAR, the vaccination program started in January 2021, which may have contributed to lowering case numbers. Micro PAR was then introduced and successfully maintained the line until May 2021, when cases started rising again due to the relaxation of policies and emerging new COVID-19 variants. Emergency PAR commenced in July 2021 resulted in a decline in the number of COVID-19 cases reported.

As mentioned earlier, the vaccination program commenced in January 2021; by the end of April 2021, 81.39% of HSGH staff had received second dose vaccination. Figure 1 shows a decreasing number of COVID-19 cases among HCWs and the general population from late January 2021. Data showed that vaccinated staff were slightly more likely to have milder disease with 97% compared with 95% in unvaccinated staff, and none had critical disease compared with 1% in unvaccinated staff, as shown in Table 1. Data also showed that hospital admission was needed in 4% of vaccinated staff and 5% of unvaccinated staff. Unfortunately, the data showed a similar outcome between vaccinated and unvaccinated staff with 99% discharged and 1% mortality.

### COVID-19 testing policy

**Figure 2:**
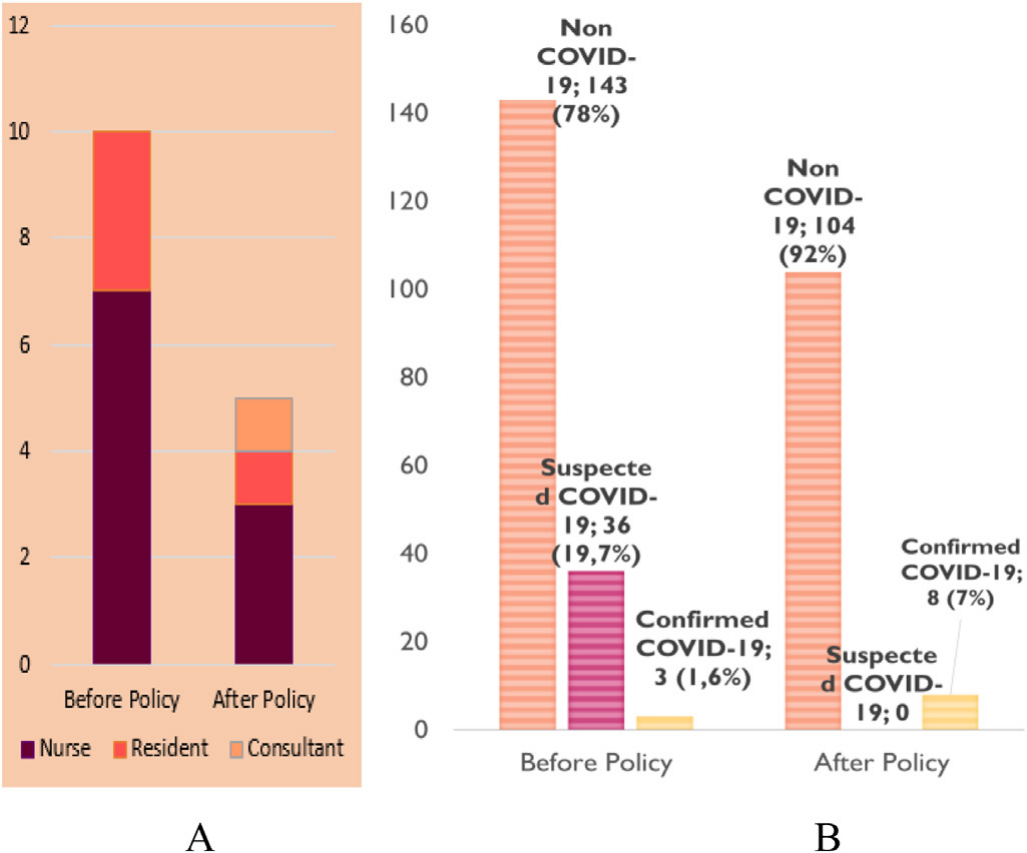
(A) Number of cases among HCW before (July-September 2020) and after (October 2020-January 2021) routine screening policy for admitted patients, (B) Number of cases among oncology patients before (July-September 2020) and after (October 2020-January 2021) routine screening policy for admitted patients

From 1 July to 2 December 2020, when PCR was only performed if the patient was suspected for COVID-19, there were 3 positive results from 36 suspected patients out of a total of 181 pediatric oncology patients. In addition, there were 10 cases among HCWs in the pediatric ward that might relate to patient transmission. After routine screening was introduced, there were 8 positive results from 121 patients admitted, all were asymptomatic, and 4 came from a boarding house. We also found that 3 patient caregivers were positive. During this period, there were 5 positive results among HCWs who did not have any relation with confirmed COVID-19 patients (Figure 2).

## Discussion

HSGH is a tertiary referral hospital with 3028 COVID-19 patients admitted up to May 2021. Hospital staff are one of the populations most vulnerable to COVID-19 infection. There are a high proportion of women among HSGH staff and their high mobility outside of the hospital explains the majority of female staff infected with COVID-19. This finding aligns with studies conducted in China by Liu et al. in the HCW population and by Xu et al. in the general population, but is contrary to other studies worldwide (Monod et al., 2021, Mutambudzi et al., 2021, Murphy et al., 2020).

Epidemiologically, most studies show that older people are at higher risk of COVID-19 and severe disease due to ACE2 receptors increasing with age (Monod et al., 2021, Mutambudzi et al., 2021). Meanwhile, Monod et al. found that early adults are also at risk due to higher transmission rates (Murphy et al., 2020). This finding aligns with our study, where most of the staff infected were 31−40 years old.

Occupation plays a large role in COVID-19 transmission and infection, even among those who work in the same place. Karlsson et al. and Mutambudzi et al. showed that patient-facing HCWs have a higher risk than non-patient-facing workers (Chung et al., 2021). Liu et al. found that most HCWs infected are nurses (Pan et al., 2021). In our study, nurses and physicians are 2 occupations at high risk of COVID-19. Our study also found a high infection rate among non-medical staff with 27% of infections. This finding could be explained by a recent study showing that infection within hospital can be transmitted from patients to staff or among the staff themselves through sharing environment and equipment (EMG - Transmission Group 2021).

Our study showed that most HSGH staff infected with COVID-19 worked in the green zone (lower risk). This finding relates to a study from Ziaquin et al., which showed a lower number of COVID-19 infections in HCWs working in fever clinics compared with those working in non-fever clinics (Pan et al., 2021). One explanation might be lower compliance and vigilance on health protocols when the awareness of the possible risk of infection is lower, indicating the importance of continuing education, especially in lower risk areas that are sometimes neglected because of the attention to COVID-19 areas.

Analysis of weekly cases reported in HSGH compared with national data showed a number of fluctuations in HSGH, compared with a steadily increasing national trend. Weekly national cases first peaked in January 2021 when the health protocol policy relaxed, with a second peak in July 2021 when the Delta variant outbreak was reported in Indonesia. In contrast, there are several peaks in the number of cases among HSGH staff due to several incidences of mass COVID-19 testing. Figure 1 shows that in the case of the Delta variant outbreak, HSGH appears to precede the national curve with the peak in June 2021. These findings indicate that COVID-19 infections are multifactorial and not only determined by occupation and workplace. Individual factors, such as sex, age and comorbidity, and external factors contribute to COVID-19 infection (Monod et al., 2021). A study in the Netherlands even showed that COVID-19 infection among HCWs tends to be due to community transmission (Parohan et al., 2021).

Despite various policies to control COVID-19 in Indonesia, Figure 1 shows a steady increase in COVID-19 cases reported. Testing and tracing were still a challenge in Indonesia due to limited health resources (Sutomo et al., 2021). Along the way, the testing and tracing protocol developed, and mass testing started to be conducted everywhere. Data also shows that public compliance to large-scale social restriction was lacking (Peraturan Presiden Republik Indonesia 2018). These factors, combined with a relaxation of health policy, may have contributed to the increasing number of cases reported in January 2021. Another contributing factor was holidays in December 2020 and New Year's Eve celebrations that invited a social crowd. Then a stricter health policy, Emergency PAR, was applied and the number of reported cases began to decrease. With a decreasing number of cases reported, health policy again began to relax, and a surge of Delta variant, Eid celebration and back to hometown tradition in May 2021 led to the second wave in Indonesia (Peraturan Presiden Republik Indonesia 2018). Borchering et al. also predicted this increasing number of cases due to the spread of the Delta variant, which is thought to be more transmissible than any other variant (Toharudin et al., 2021). Again, stricter health policy was applied, and the curve began to fall in July 2021.

Work-from-home and work-from-office policies played a significant role in controlling the pandemic by limiting crowds and promoting social distancing in the office (Vyas and Butakhieo, 2021, Wang et al., 2021). Bracis et al. found that the risk of getting COVID-19 is higher among those who work in an office (Wang et al., 2020). The policy of restricting staff from going out of town also limited the transmission of COVID-19 from one place to another. Testing and tracing also played a role in the number of cases reported. These are the most powerful public health interventions available. The less testing and tracing conducted, the lower possibility of cases being reported (Peraturan Presiden Republik Indonesia 2021). Andriani showed that, at the start of the pandemic, the testing rate in Indonesia was only 136 per 1 million people compared with Malaysia with 2988 and Singapore with 16 203 per 1 million people (Sutomo et al., 2021). Inadequate testing might result in underestimating the true number of COVID-19 cases, and therefore, mass screening also plays a significant role in detecting cases (Vyas and Butakhieo, 2021). In HSGH, routine mass testing was held several times a year, especially when clusters were reported in some wards; this explains the variations in cases reported, with several peaks among HSGH staff. This study also showed that routine PCR screening for patients admitted to hospital might be necessary to protect the hospital. Nevertheless, public policy, such as the lockdowns in several countries or social restriction in Indonesia, is effective in lowering the number of daily cases reported (Jarvis et al., 2021, Karlsson and Fraenkel, 2020, Kementerian Kesehatan Republik Indonesia 2020, Sutomo et al., 2021).

Our study showed that vaccination lowered the incidence of cases and severe disease among HSGH staff, but there were vaccination breakthrough cases. There were 6 deaths within the study period. Among them, 3 were unvaccinated, 2 were partially vaccinated and 1 was fully vaccinated but with a chronic hematology disorder as a comorbidity before COVID-19 infection. Four had comorbidities of third-semester pregnancy, hypertension, diabetes and older age; only 1 staff member without comorbidity, but unvaccinated, suffered critical condition leading to death. The 3 vaccinated staff were infected during the second outbreak of COVID-19 in Indonesia when the infection and hospital admission rate was high and the Delta variant was spreading. All 19 of the staff testing positive for COVID-19 in June 2021, when whole-genome sequencing was performed, were infected with the Delta variant. The Delta variant is reportedly resistant to vaccine, which might explain infection even in the fully vaccinated; however, vaccination has proved to have a protective effect with those vaccinated experiencing milder disease compared with those unvaccinated, even with the Delta variant (Peraturan Presiden Republik Indonesia 2018).

Our study found that 28 (1.7%) staff were reinfected with COVID-19. West et al. stated that reinfection might occur in high-risk populations, especially those with comorbidities, older people and those working in close contact. HCWs are among the high-risk populations for reinfection^40^. One study found that 6 months after the second dose, antibody titers start to decrease and put people at risk; the third dose of vaccine is proven to boost immunogenicity against COVID-19 (World Health Organization Headquarters 2021).

This study has several limitations. First, the study did not record the antibody SARS-CoV-2 titer among hospital staff after vaccination. Second, routine testing for screening patients and caregivers was not performed hospital-wide. Third, whole-genome sequencing was only performed on those testing positive in June 2021, which may not reflect the prevalence of the Delta variant among HCWs in HSGH across the study period.

In conclusion, our study found a trend in physicians and nurses being a profession with a high number of COVID-19 infections. The inconsistent fluctuation between weekly cases in the HSGH and nationally, a higher infection rate in the green zone, and the age range of those infected showed that COVID-19 infection is multifactorial. Stricter health protocols, the SAR program, routine screening for patients admitted to hospital and their caregivers, and continuing education to HCWs is needed to protect the frontline workers in the COVID-19 pandemic.

## Funding

No financial support was provided relevant to this article

## Ethical Approval

This study has been approved by the ethics committee of Hasan Sadikin General Hospital number LB.02.01/X.6.5/329/2020.

## Authorship and manuscript preparation

RA, SJ, EVF performed contact tracing, data collection and participated in manuscript preparation; DP, BA, DKT, MS, LL collected and processed samples; RW, YH, AR, IAR assisted in study design and review of the manuscript.

## Declaration of Competing Interest

All authors report no conflict of interest relevant to this article

## References

[bib0001] Abohamr SI, Aldossari MA, Alaklobi FA, Amer HA, Alzarzour SH, Abdelhamid SW (2020). Clinical characteristics and in-hospital outcome of medical staff infected with COVID-19 in Saudi Arabia: A retrospective single-center study. Saudi Med J.

[bib0002] Alexandar S, Ravisankar M, Kumar RS, Jakkan K. (2021). A Comprehensive Review on Covid-19 Delta variant A Comprehensive Review on Covid-19 Delta variant.

[bib0003] Andriani S (2020). Effectiveness of Large-Scale Social Restrictions (PSBB) toward the New Normal Era during COVID-19 Outbreak: a Mini Policy Review. J Indones Heal Policy Adm.

[bib0004] Borchering RK, Viboud C, Howerton E, Smith CP, Truelove S. (2021). Modeling of Future COVID-19 Cases, Hospitalizations, and Deaths, by Vaccination Rates and Nonpharmaceutical Intervention Scenarios. Morb Mortal Wkly Rep CDC.

[bib0005] Bracis C, Burns E, Moore M, Swan D, Reeves DB, Schiffer JT (2021). Widespread testing, case isolation and contact tracing may allow safe school reopening with continued moderate physical distancing: A modeling analysis of King County, WA data. Infect Dis Model [Internet].

[bib0006] Bradshaw WJ, Alley EC, Huggins JH, Lloyd AL, Esvelt KM. (2021). Bidirectional contact tracing could dramatically improve COVID-19 control. Nat Commun.

[bib0007] Burrer SL, de Perio MA, Hughles MM, Kuhar DT, Luckhaupt SE, McDaniel CJ (2020). Characteristics of Health Care Personnel with COVID-19 - United States, February 12-April 9, 2020. Centers Dis Control Prev.

[bib0008] Chung SC, Marlow S, Tobias N, Alogna A, Alogna I, You SL (2021). Lessons from countries implementing find, test, trace, isolation and support policies in the rapid response of the COVID-19 pandemic: a systematic review. BMJ Open.

[bib0009] EMG - Transmission Group. COVID-19 Risk by Occupation and Workplace. 2021;(February)

[bib0010] Gitiyarko V. PSBB Hingga PPKM, Kebijakan Pemerintah Menekan Laju Penularan Covid-19. 2021 Aug 1; Available from: https://kompaspedia.kompas.id/baca/paparan-topik/psbb-hingga-ppkm-kebijakan-pemerintah-menekan-laju-penularan-covid-19

[bib0011] Iavicoli S, Boccunii F, Buresti G, Gagliardiid D, Persechino B, Valenti A (2021). Risk assessment at work and prevention strategies on COVID-19 in Italy. PLoS One [Internet].

[bib0012] Jagadeesh Kumar V, Sowpati DT, Munigela A, Banu S, Siva AB, Sasikala M (2021). Clinical outcomes in vaccinated individuals hospitalized with Delta variant of SARS-CoV-2. medRxiv [Internet].

[bib0013] Jarvis CI, Gimma A, van Zandvoort K, Wong KLM, Abbas K, Villabona-Arenas CJ (2021). The impact of local and national restrictions in response to COVID-19 on social contacts in England: a longitudinal natural experiment. BMC Med.

[bib0014] Karlsson U, Fraenkel C-J. (2020). Covid-19: risks to healthcare workers and their families Mistakes. BMJ.

[bib0015] Kementerian Kesehatan Republik Indonesia (2020). Pedoman Pencegahan dan Pengendalian Corona Virus deases (Covid-19). Kementrian Kesehat [Internet].

[bib0016] Kementerian Kesehatan Republik Indonesia. Peta Sebaran Covid-19 Indonesia [Internet]. 2021 [cited 2021 Jul 25]. Available from: https://covid19.go.id/peta-sebaran

[bib0017] Liu J, Ouyang L, Yang D, Han X, HanCao Y, Alwalid O (2021). Epidemiological, clinical, radiological characteristics and outcomes of medical staff with COVID-19 in Wuhan, China: Analysis of 101 cases. Int J Med Sci.

[bib0018] Mousten-Helms IR, Emborg H-D, Nielsen J, Nielsen KF, Krause TG, Molbak K (2021). Vaccine effectiveness after 1st and 2nd dose of the BNT162b2 mRNA Covid-19 Vaccine in long-term care facility residents and healthcare workers – a Danish cohort study. BMJ.

[bib0019] Monod M, Blenkinsop A, Xi X, Hebert D, Bershan S, Tietze S (2021). Age groups that sustain resurging COVID-19 epidemics in the United States. Science (80-).

[bib0020] Mutambudzi M, Niedwiedz C, Macdonald EB, Leyland A, Mair F, Anderson J (2021). Occupation and risk of severe COVID-19: Prospective cohort study of 120 075 UK Biobank participants. Occup Environ Med.

[bib0021] Murphy K, Williamson H, Sargeant E, McCarthy M. (2020). Why people comply with COVID-19 social distancing restrictions: Self-interest or duty?. Aust New Zeal J Criminol.

[bib0022] Pan H, Wu Q, Zeng G, Yang J, Jiang D, Deng X, et al. Immunogenicity and safety of a third dose, and immune persistence of CoronaVac vaccine in healthy adults aged 18-59 years: interim result from a double-blind, randomized, placebo-controlled phase 2 clinical trial. 2021;

[bib0023] Parohan M, Yaghoubi S, Seraji A, Javanbakht MH, Sarraf P, Djalali M. (2021). Risk factors for mortality in patients with Coronavirus disease 2019 (COVID-19) infection: a systematic review and meta-analysis of observational studies. Aging Male [Internet].

[bib0024] Peraturan Presiden Republik Indonesia. Undang Undang Nomor 6 tahun 2018 tentang Kekarantinaan Wilayah. 2018;31–4. Available from: https://jdih.bsn.go.id/produk/detail/?id=730&jns=2

[bib0025] Sinuhaji J. 10 Negara dengan Kasus Aktif Covid-19 Tertinggi di Dunia, Indonesia Urutan ke-5. Pikiran Rakyat [Internet]. 2021; Available from: https://www.pikiran-rakyat.com/internasional/pr-012329069/10-negara-dengan-kasus-aktif-covid-19-tertinggi-di-dunia-indonesia-urutan-ke-5

[bib0026] Sinuhaji J. Hari Ini Kasus Kematian Covid-19 Indonesia Tembus 100.000 Orang, Peringkat ke-12 di Dunia. Pikiran Rakyat [Internet]. 2021; Available from: https://www.pikiran-rakyat.com/nasional/pr-012335100/hari-ini-kasus-kematian-covid-19-indonesia-tembus-100000-orang-peringkat-ke-12-di-dunia

[bib0027] Sutomo S, Sagala S, Sutomo B, Liem W, Al Hamid H (2021). Strengthening the Strategic and Operational Response for Reducing COVID-19 Transmission in Indonesia.

[bib0028] Toharudin T, Pontoh RS, Caraka RE, Zahroh S, Kendogo P, Sijabat N (2021). National vaccination and local intervention impacts on covid-19 cases. Sustain.

[bib0029] Vyas L, Butakhieo N. (2021). The impact of working from home during COVID-19 on work and life domains: an exploratory study on Hong Kong. Policy Des Pract [Internet]..

[bib0030] Wang C, Wang D, Abbas J, Duan K, Mubeen R. (2021). Global Financial Crisis, Smart Lockdown Strategies, and the COVID-19 Spillover Impacts: A Global Perspective Implications From Southeast Asia. Front Psychiatry.

[bib0031] Wang D, Hu B, Hu C, Zhu F, Liu X, Zhang J (2020). Clinical Characteristics of 138 Hospitalized Patients with 2019 Novel Coronavirus-Infected Pneumonia in Wuhan, China. JAMA - J Am Med Assoc.

[bib0032] World Health Organization. WHO Coronavirus Disease (COVID-19) Dashboard [Internet]. 2021 [cited 2021 Feb 22]. Available from: https://covid19.who.int/

[bib0033] World Health Organization Headquarters (2021). Clinical management Clinical management Living guidance COVID-19. World Heal Organ [Internet].

